# Experience beyond the learning curve of transanal total mesorectal excision (taTME) and its effect on the incidence of anastomotic leak

**DOI:** 10.1007/s10151-020-02160-6

**Published:** 2020-02-28

**Authors:** A. Caycedo-Marulanda, C. P. Verschoor

**Affiliations:** 1 Department of Surgery, Health Sciences North, 65 Larch St, Sudbury, ON P3E 1B8 Canada; 2 Health Sciences North Research Institute, Sudbury, ON Canada; 3 Department of Surgery, Northern Ontario School of Medicine, Sudbury, ON Canada

**Keywords:** Rectal cancer, Transanal total mesorectal excision, Anastomotic leak, Learning curve, Single team

## Abstract

**Background:**

The most important advancement in the surgical management of rectal cancer has been the introduction of total mesorectal excision (TME). Technical limitations to approaching mid and distal lesions remain. The recently described transanal TME makes it possible to minimize some of the difficulties by improving access. Anastomotic leak is a persistent concern after colorectal surgery no matter what technique is used. The objective of this study was to explore the impact of experience on the incidence of anastomotic leak after transanal TME. Secondary endpoints were local recurrence and margin status.

**Methods:**

A retrospective cohort study was conducted over a period of 3 years at a tertiary care center in Northern Ontario with high volume of rectal cancer patients. The initial 100 consecutive patients with rectal neoplasia who had transanal TME surgery were included. All cases were performed by a single team. The main outcome assessed was the incidence of anastomotic leak beyond a pre-determined learning curve, as previously established in the literature. For statistical analysis, associations between patient characteristics and outcomes were estimated using ordinary least squares and logistic regression.

**Results:**

Six cases of anastomotic leak occurred over the course of the study, the last of which occurred in the 37th patient. Relative to a baseline anastomotic leak rate of 7.8%, cumulative sum (CUSUM) analysis indicated that a 50% improvement in risk occurred at trial 50 of 85 patients that had an anastomosis performed. Two patients developed local recurrence during the study period. No correlation between learning curve and oncologic outcomes was identified.

**Conclusions:**

Proficiency is likely to have a positive effect on the 30-day occurrence of anastomotic leak. Larger studies are required to explore the impact of experience on local recurrence.

## Introduction

The most important advancement in the surgical management of rectal cancer has been the introduction of total mesorectal excision (TME) [1, 2]. Patients with locally advanced disease benefit from the addition of multimodality therapy with chemoradiation therapy [3]. Evidence indicates that surgical difficulties are influenced by surgeon’s skills and patients’ anatomical and clinical factors. Particularly unfavorable characteristics include male sex, obesity, low tumor location, tumor size, and narrow pelvis [4, 5]. There are technical challenges associated with both open and laparoscopic approaches; nevertheless, TME has been shown to have a positive impact on local recurrence and curative rates [6, 7]. More recently, a bottom-up approach has been described, known as the transanal TME (taTME) [8], which has gained increasing attention from the surgical community. In theory, taTME mitigates some of the technical difficulties of operating in the mid and distal rectum because of perpendicular division of the mesorectum and a more precise dissection due to improved access to the target. Multiple publications have accrued evidence supporting the procedure [9, 10]. However, safe implementation has demonstrated to be lengthy and challenging despite the wide availability of surgical workshops and the development of training pathways [11–13].

Lee et al. have reported on proficiency and the likelihood of achieving it after 45–51 cases [14]. Experience and volume seem to have a significant impact on most of the major short-term postoperative outcomes after taTME surgery, including oncologic results and postoperative complications such as anastomotic leak (AL) [14, 15]. A persistent concern is the potential marginal increase of AL related to the procedure, which, according to recent literature, could be up to 12–15% overall and close to 8% in the 30 days following surgery [16, 17]. Anastomotic failure after the construction of a colorectal anastomosis is associated with significant morbidity and mortality; the risk of permanent stoma after AL is more than 60% and oncologic results, including overall survival, disease-free survival, and cancer-specific survival, are definitively less favorable [18]. In a retrospective analysis, Kang et al. identified the overall AL rate after low anterior resection (open and laparoscopic) to be 13.7%, and a higher overall mortality in those cases was also identified in this study [16, 19]. According to the most recent publication by the international registry, on 1594 patients, the main risk factors for AL after taTME surgery included male sex, obesity, tumors > 25 mm, excessive intraoperative blood loss, manual anastomosis, and prolonged perineal operative time [17].

The objective of this study was to determine the effect of experience and proficiency on the incidence of AL after taTME surgery in the single-team setting. Secondary outcomes included the incidence of complete/near complete pathological specimens, margin positivity, intra- and postoperative complications, readmission, local recurrence rate, conversion, and length of stay.

## Materials and methods

### Settings and patients

The study cohort consisted of the initial 100 consecutive patients who underwent taTME surgery at a tertiary care center in Northern Ontario [Health Sciences North (HSN), over a period of 3 years (June 2015–July 2018)]. All patients had either histopathologic proof of neoplasia with or without confirmation of malignancy or large lesions with radiologic assessment reporting high suspicion of invasion, therefore, warranting radical excision. A prospectively collected and maintained database was queried for this study. We preferentially included patients with tumors located in the mid-low rectum. All cases had colonoscopic and radiologic staging by computed tomography (CT) scan of the chest, abdomen, and pelvis as well as pelvic magnetic resonance imaging (MRI). Procedures were performed by a single team composed of one sub-specialty trained colorectal surgeon, a surgical assistant, and two operating room nurses. Approval of the study protocol was obtained from the institutional ethics review board.

### Surgical technique

A brief description of our previously published surgical technique [20]. Prior to surgery, all patients receive mechanical bowel preparation. Patients are set on the Pink Pad™ positioning device system (New Kensington, PA, USA) in lithotomy position, under general anesthesia. Prophylactic antibiotics and anticoagulants are administered. Under aseptic conditions, a regular laparoscopic approach is used to perform an oncologic dissection of the left colon including release of the splenic flexure. Our preference is the medial-to-lateral approach. This is ensued by dissection of the upper rectum to the level of the peritoneal reflection or lower if possible. Once the top dissection is completed, a Lonestar® retractor (Trumbull, CT, USA) is placed to efface the anal canal, and the TAMIS ® levator sling (Rancho, Santa Margarita, CA, USA) is introduced into the anal canal. A closed purse string is created with 0-prolene ® below the level of the tumor, and the purse string is secured air tight, followed by a thorough washout. The rectal wall is incised circumferentially with monopolar cautery and full-thickness division of the rectal wall is performed. The mesorectal plane is then dissected from the bottom-up until the rendezvous occurs. Occasionally, it is necessary for the surgeon to go back and forth from the top to the bottom. A special attention is placed on preserving the integrity of the surrounding structures. A decision is made regarding transanal versus transabdominal extraction. Finally, in cases where reconstruction is feasible, this is carried out with a transanal circular stapler versus a hand-sewn coloanal anastomosis and the majority of these patients have a diverting loop ileostomy installed.

### Outcomes

The main objective of this study was to identify the occurrence of AL during the first 30 days after taTME surgery. As experience was acquired, we looked at its effect on the incidence of leakage. AL was defined as evidence of pelvic infection determined by any combination of the following: pelvic pain, purulent secretion per rectum, increased withe blood cell count, and confirmation of anastomotic dehiscence by axial imaging and/or endoscopic evaluation demonstrating disruption of the anastomosis. Secondary outcomes included completeness of the mesorectum and margin positivity, intraoperative complications, 30-day perioperative outcomes including morbidity, conversion, 30-day readmission, and length of stay. Local recurrence was also considered. Quality of the mesorectum and margin positivity was assessed by the institutional pathologists following the Nagtegaal classification [21].

### Statistical analysis

Patient characteristics included age, sex, body mass index (BMI), tumor height, TNM stage (0–4 or not determined), neoadjuvant chemotherapy and radiotherapy (yes/no), OR duration, and anastomosis type (circular stapler, hand sewn, or none) (Table 1). Outcome measures included AL (yes/no), mesorectal resection quality (incomplete or complete/near complete), tumor margins (positive or negative), 30-day postoperative complications (yes or no), intraoperative complications (yes/no), 30-day readmission (yes/no), conversion (yes/no) length of stay, and local recurrence (yes/no) (Table 2). Characteristics and outcomes were summarized as mean ± standard deviation (minimum/maximum) or count.

**Table 1:** Summary of patient characteristics

	*N*	Mean ± SD (min/max)
Age, years	100	64 ± 10.8 (39.6/85)
Body-mass index	100	27.6 ± 6.5 (16.8/50.4)
Tumour height	100	6.17 ± 2.57 (0/13)
Operation duration	100	5.09 ± 1.19 (0.25/8.02)

Value	Count
Sex	
Female	32
Male	68
Stage	
T (primary tumour)	
2	22
3	64
4	8
ND	4
NA's	2
N (regional lymph nodes)	
0	44
1	45
2	11
M (distant metastasis)	
0	91
1	8
NA's	1
Neoadjuvant therapy	
Chemotherapy	
No	36
Yes	64
Radiotherapy	
No	32
Yes	68
Anastomosis type	
Circular stapler	70
Hand sewn	15
None	15

*NA* missing data, *ND* not determined, *SD* standard deviation, *min/max* minimum/maximum, *N* sample size

**Table 2:** Summary of recorded outcomes

Value	*N*
Anastomotic leak^a^	
No	94
Yes	6
Quality (surgical specimen)	
Complete	83
Near complete	17
Margins	
Circumferential	
Negative	97
Positive	3
Distal	
Negative	100
Complications	
Postoperative	
No	66
Yes	34
Intraoperative	
No	98
Yes	2
Readmission	
No	89
Yes	11
Conversion	
No	100
Local reoccurrence	
No	98
Yes	2

^a^‘No’ anastomotic leak includes those patients in which no anastomosis was performed (*n* = 15). These individuals were removed prior to regression and CUSUM analyses

Associations between our patient characteristics and outcomes were estimated using ordinary least squares and logistic regression. Univariate models were performed in addition to multivariable models that were adjusted for sex, BMI, and tumor height; these covariates were selected a priori. Only those associations for the outcomes AL and length of stay are presented as all other outcomes either lacked an appreciable number of events for the levels recorded or did not exhibit associations with any of the patient characteristics. For AL specifically, we removed those patients that did not have an anastomosis performed (*n* = 15) prior to analysis. For all regression analyses, *p* < 0.05 was considered significant.

To monitor improvement in our surgical procedure for the outcome of AL, we performed a non-risk adjusted cumulative sum (CUSUM) chart analysis [22] using the R package ‘cusum’. This approach is effective for assessing the learning curve for a repeated procedure as it monitors the occurrence of a binary outcome over time and indicates when the rate of occurrence changes significantly. For our analysis of AL, the baseline risk was set to 7.8%, as previously reported by Penna et al. [23], and the threshold was set to detect a 50% improvement from the baseline risk. As with our regression analyses, patients that did not have an anastomosis performed were removed prior to analysis. All analyses were performed in R v3.6.

## Results

The 100 patients in our sample were mostly male (68%), with a mean age of 64 ± 10.8 years, a mean body mass index (BMI) of 27.6 ± 6.5 kg/m^2^, and mean tumor height of 6.17 cm ± 2.57 cm (0/13) (Table 1). The leak rate of anastomotic reconstruction within 30 days was 7.1% (85 patients with anastomosis and 6 with AL). No incomplete specimens were identified. Three percent of circumferential tumor margins were positive (cases 10, 59, and 94); no distal margins were positive. Intraoperative complications occurred in 2 patients: an early case with presacral bleeding and a later case with splenic injury requiring a splenectomy, performed in a second intervention. There were no intraoperative conversions. The 30-day readmission rate was 11% (Table 2).

Of the outcomes recorded, circumferential and distal margins, intraoperative complications, and conversion exhibited less than 5% incidence (Table 2). The remaining outcomes were regressed against the patient characteristics age, sex, BMI, tumor height, neoadjuvant therapy, and anastomosis type in both a univariate and multivariable fashion; other characteristics listed in Table 1 were either exhibited too little variance or were not of enough clinical interest to warrant performing regression analysis. Following univariate regression, length of stay was the only secondary outcome to exhibit a significant association with patient characteristics; hence, only associations with AL (primary outcome) and length of stay are reported (Table 3). In both univariate and multivariable analyses, AL was not associated with any patient characteristics. Males had a significantly longer length of stay than females (beta [95% CI] = 3.2 days [1.4, 4.9], *p* < 0.001) after adjusting for BMI and tumor height, while tumor height was inversely correlated (− 0.38 cm [− 0.69, − 0.07]) after adjusting for sex and BMI (Table 3).

**Table 3:** Associations between participant characteristics and the outcomes anastomotic leak and length of stay

Variable	Level	Anastomotic leak (OR [95% CI], *p* value)		Length of stay (beta [95% CI], *p* value)	
		Univariate model	Multivariable model	Univariate model	Multivariable model
Age, years	-	1.01 [0.93–1.09], 0.887	1.03 [0.94–1.12], 0.551	0.06 [− 0.02 to 0.13], 0.134	0.01 [− 0.07 to 0.09], 0.797
Sex	Female	Ref	Ref	Ref	Ref
	Male	0.46 [0.08–2.65], 0.366	0.4 [0.07–2.34], 0.288	**2.9 [1.19 to 4.61], 0.001**	**3.17 [1.44 to 4.89], 0.0004**
Body-mass index, kg/m^2^	–	1.02 [0.89–1.15], 0.725	1.02 [0.88–1.15], 0.777	0.02 [− 0.11 to 0.15], 0.801	0.02 [− 0.11 to 0.14], 0.806
Tumour height, cm	–	1.09 [0.77–1.57], 0.621	1.12 [0.78–1.63], 0.546	-0.29 [− 0.62 to 0.03], 0.075	− **0.38 [**− **0.69** to − **0.07], 0.018**
Neoadjuvant (chemotherapy)	No	Ref	Ref	Ref	Ref
	Yes	0.52 [0.09–2.97], 0.441	0.48 [0.07–2.98], 0.42	0.75 [− 0.98 to 2.48], 0.393	0.51 [− 1.15 to 2.18], 0.542
Neoadjuvant (radiation)	No	Ref	Ref	Ref	Ref
	Yes	0.44 [0.08–2.5], 0.331	0.42 [0.06–2.61], 0.333	0.51 [− 1.28 to 2.3], 0.572	0.03 [− 1.7 to 1.76], 0.97
Anastomosis type	Circular stapler	Ref	Ref	Ref	Ref
	Hand sewn	NA	NA	0.68 [− 1.71 to 3.07], 0.573	− 0.19 [− 2.85 to 2.47], 0.89
	None	NA	NA	0.55 [− 1.84 to 2.94], 0.65	− 0.9 [− 3.43 to 1.63], 0.482

Associations with anastomotic leak were estimated by logistic regression, and the odds ratio (OR), 95% confidence interval (CI), and *p* value were presented. Associations with length of stay were estimated by linear regression, and the regression coefficient (beta), 95% CI, and *p* value are presented. Multivariable models were adjusted for sex, body mass index, and tumor height. Significant (*p* < 0.05) associations are in bold

*Ref* reference level, *NA* not enough events were available to reliably calculate confidence intervals

**Fig. 1:**
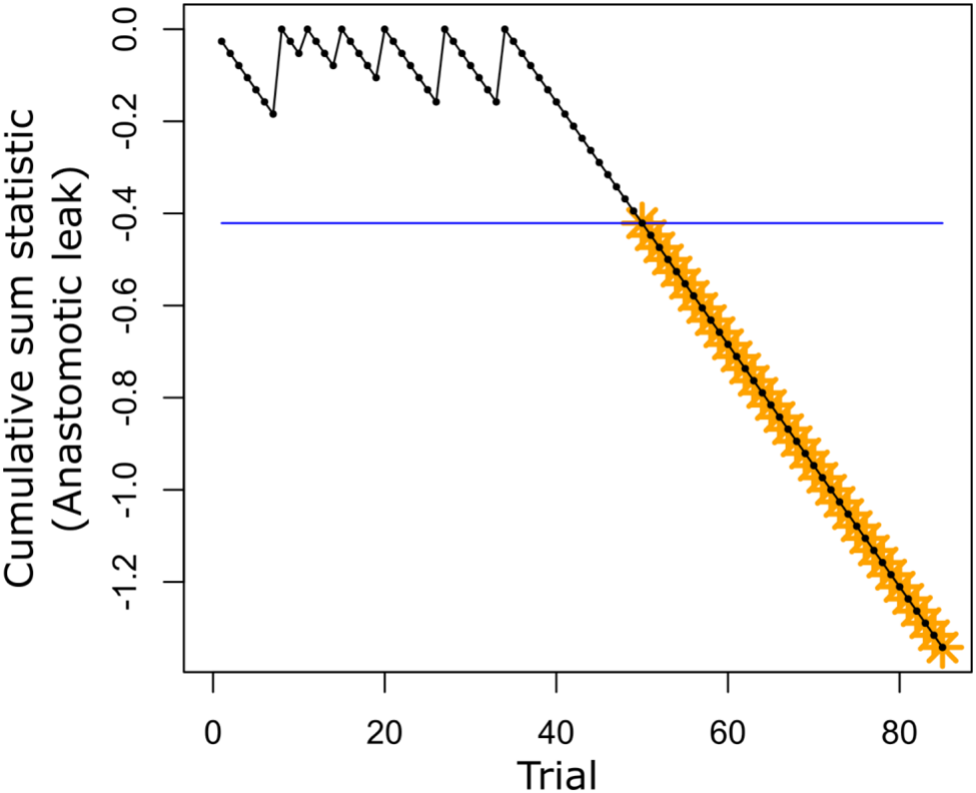
Cumulative sum (CUSUM) analysis to detect improvement in the occurrence of anastomotic leak. Baseline risk was set to 7.8% and the threshold was set to detect a 50% improvement in the outcome rate. For this analysis, the threshold was crossed at trial number 50

A primary objective of our study was to determine the point at which surgeons significantly improve their proficiency in taTME, using AL as our benchmark outcome. Six cases of anastomotic leak occurred, the last on trial 37. Relative to a baseline AL rate of 7.8% (23), CUSUM analysis indicated that a 50% improvement in risk occurred at trial 50 of 85 patients that had an anastomosis performed (Fig. 1). Follow-up in our cohort ranged from 1639 to 491 days. During the study period, we only identified two cases of local recurrence (cases 13 and 86). For both trials, specimens after surgery were deemed complete with negative margins; thus, a recurrence rate of 2% for the study period is reported. The first patient, a healthy 42-year-old woman, had a distal tumor staged as T2N0 disease: no neoadjuvant chemoradiation was offered and sphincter preservation was possible. The second patient was a 77-year-old man with T3N1 disease who received neoadjuvant chemoradiation. After surgery, he had a prolonged ileus that lengthened his hospital stay to 11 days. There were no intraoperative complications in either case. Recurrences were identified 553 and 484 days after the index operation, respectively, and the second patient eventually succumbed to his disease. The recurrence pattern in both cases was unifocal and occurred over a year after the initial resection.

## Discussion

Newer surgical approaches for the management of rectal cancer, including taTME have recently been introduced [24]. Although there is evidence of acceptable short-term outcomes of taTME [9, 17, 25], concerns and criticism regarding the safety and generalizability of the procedure have been voiced [12, 26, 27]. The publication by Lee et al. [14] estimated that to become proficient in taTME, 45 procedures are required if only low anterior resections are considered and 51 cases if abdominoperineal resections are included. In another publication exploring the learning curve of taTME [28], the authors focused on operative time and major postoperative morbidity. They showed competency was achieved after 40 cases and indicated improved patient-related clinical outcomes, since operative time was identified as a poor surrogate of surgical quality. The average leak rate reported in their 138 patients was 13.8%; however, this dropped from 27.5% in the first 40 patients to under 5% in the subsequent cases. Interestingly, the authors included lesions between 0 and 15 cm and the average location was 7.3 cm. In our center, we have elected to reserve taTME for lesions in the mid-low rectum with an average height of 6.17 cm from the anal verge. The international registry provides the largest number of patients in any publication so far, and the early incidence of AL is 7.8%; however, this needs to be interpreted with caution, since the registry is a voluntary collaboration and under-reporting is possible [17].

AL and postoperative complications have an unequivocally deleterious impact on patients’ functional and oncological outcomes [18, 29], hence the importance of minimizing their incidence. taTME has proven to be difficult to adopt and challenging to become proficient at; the risk of experiencing leaks must be lowered to really accept the technique as advantageous. We encountered an average AL incidence of 7.1%, representing 6 out of 85 patients in whom an anastomosis was created. More importantly, events occurred within the initial 37 cases, well within the described learning curve [14, 28]. As experience grew and proficiency was acquired, our AL incidence dropped to 0 after case number 38. We performed univariate and multivariate analyses and no specific correlations were identified between any specific factor and AL, although this may be related to the relatively few number of events. We found that male sex and increased BMI were significant factors for an increased length of stay.

Lee et al. considered completeness of the mesorectum as the most important measure of surgical quality [14]; acceptable and unacceptable rates of good-quality TME were defined in accordance to the ACOSOG Z6051 trial (laparoscopic unacceptable rate was 81.7% and open acceptable rate was 86.9%) [30]. In their series of 87 consecutive patients, a high-quality TME was identified in 95% of cases, negative circumferential radial margin (CRM) was 98%, and distal rectal margin (DRM) was 99%. Based on those results, the authors concluded that a range of 45–51 taTME cases is required to reach an acceptable incidence of high-quality TME. Simultaneously, de Lacy’s group published on the largest series from a single center [31]. Their study included 186 consecutive patients with a complete TME in 97.3% of cases; positive CRM (≤ 1 mm) and distal resection margin (DRM) (≤ 1 mm) were 8.1% and 3.2%, respectively. Finally, Kodeman et al. from the Netherlands [28], who reported on their estimated learning curve as well, obtained high-quality TME in 99% of cases, positive CRM (0%), and DRM was 1.4%. All these results come from three of the most experienced groups in taTME surgery in the world and are definitively above the range of acceptability. Our results are comparable to those above; however, the most relevant difference is that in our center, all cases were performed using a single-team approach (see Table 4). The incidence of local recurrence after taTME has become a point of central concern since the issuing of the Norwegian moratorium [26]. Its relevance has been perfectly illustrated by Atallah et al. in a recent editorial [32]. Our first local recurrence occurred in a healthy 42-year-old female who underwent surgery within the learning curve period. In this case, the patient developed pelvic pain associated with an increased carcinoembryonic antigen level and recurrent disease was found on imaging to be inside the sacrum. After biopsy, the patient underwent further surgery and radiation therapy. The second case of local recurrence occurred in a 77-year-old male after proficiency was reached. Recurrent disease was inside the pelvis encasing the anastomosis, and the patient was debilitated and only received palliative management. We did not identify multiple focal recurrence in either of the patients. Our local recurrence rate and pattern are in keeping with the recent publication of the long-term results published by the Netherlands group [26].

**Table 4:** Representation of the variables of a high-quality TME after taTME as reported by different authors

	Complete/near complete (%)	CRM (%)	DRM (%)
Koedam et al. 2018 [28]	99	1.40	0
Lee et al. 2018 [14]	99	2	1
Lacy et al. [9]	97.30	8.10	3.20
Caycedo-Marulanda et al. 2020 (this article)	100	3	0

*CRM* circumferential radial margin, *DRM* distal radial margin

The difficulties surrounding taTME implementation have been previously reported by various authors [11, 12, 33]. Expert consensus recommends having the previous experience on transanal surgery, having two surgeons training at high-volume centers followed by mentorship and online education [34]. In the past, we emphasized that a single-team (surgeon) taTME program is feasible [35]; however, we also stated that this does not constitute justification nor endorsement for liberal single-surgeon taTME implementation without a careful evaluation of the needs and availability of resources. The latter statement needs to be seriously considered by any potential operator and/or institution entertaining the idea of adopting this technique. While it is difficult to predict when a surgeon becomes proficient in a determined surgical technique, considering our results and those obtained by others, it is fair to assume that the incidence of AL and its consequences can be seriously diminished once proficiency is achieved. It is necessary to implement very rigorous standards when adopting a unique and complex technique such as taTME.

This study has limitations: it was conducted in a single center with dedicated experience on the procedure; therefore, generalizability of the results may pose a challenge. Additionally, other patient-related factors previously reported to have an impact on AL, such as diabetes and smoking, were not part of the analysis; therefore, their effect on this study is not known.

## Conclusions

Adequate training, high volume, and experience may potentially play a significant role on the incidence of complications, especially incidence of AL and their consequences, after taTME surgery. Proficiency is likely acquired after 45–51 or more cases, and this has a positive effect on the 30-day occurrence of anastomotic leak. We did not identify a correlation between local recurrence and experience; to explore this effect, it is possible that larger studies are required. Implementation of the technique is challenging and the learning curve in a single-team setting is likely more prolonged than it is for a double-team approach.
